# Trigeminal interfascicular neurolysis (nerve combing) for refractory recurrent neuralgia in multiple sclerosis

**DOI:** 10.3171/2020.5.FOCVID203

**Published:** 2020-10-01

**Authors:** Paolo Ferroli, Ignazio G. Vetrano, Francesco Acerbi, Gabriella Raccuia, Marco Schiariti, Paolo Confalonieri, Luisa Chiapparini, Morgan Broggi

**Affiliations:** 1 Neurosurgical Unit II, Department of Neurosurgery,; 2 Unit of Neuroimmunology and Neuromuscular Diseases, and; 3 Neuroradiology Department, Fondazione IRCCS Istituto Neurologico Carlo Besta, Milan, Italy

**Keywords:** microvascular decompression, interfascicular neurolysis, multiple sclerosis, nerve combing, trigeminal neuralgia

## Abstract

In multiple sclerosis (MS) patients, trigeminal neuralgia (TN) represents a challenging syndrome to treat, often refractory to medical therapy and percutaneous techniques. Despite the frequent lack of a neurovascular conflict, the trigeminal nerve’s axons are often damaged, with the myelin sheath permanently degenerated, thus explaining the difficulty in treating TN in MS.

The authors illustrate trigeminal interfascicular neurolysis (the combing technique) to control refractory recurrent TN in MS: the nerve is longitudinally divided along its fibers from the root entry zone, determining good pain relief.

The video can be found here: https://youtu.be/o1XksPW5fMY

## Transcript

**0:23 Case Presentation.** We present a case of a 58-year-old woman affected by recurrent, drug-resistant trigeminal pain and multiple sclerosis diagnosed in 2003.^1^ Two previous percutaneous balloon compressions and a microvascular decompression obtained only transient pain relief. MR did not reveal any new vascular cross compression, and surgical exploration of the trigeminal root with interfascicular neurolysis was proposed.^2^

**0:52 Neuroimaging.** The preoperative T2 and T1 axial MR showed multiple demyelinating lesions. The red arrow points at plaque into the pons, along the trigeminal intrapontine fibers; the blue arrow shows a vessel running close to the trigeminal nerve (yellow arrow).

**1:13 Surgical Position.** The patient is positioned supine with the head elevated and rotated toward the contralateral side; the vertex is then gently depressed inferiorly, to create an adequate working corridor between the head and the shoulder.^3^

**1:28 Skin Incision.** The anatomical landmarks are shown: the transverse and the sigmoid sinuses are drawn in blue, along with the mastoid tip and the zygomatic arch. A vertical skin incision of 5 cm is drawn in yellow and centered over the craniectomy (dotted blue).

**1:48 Retrosigmoid Mini-Craniectomy.** The previous retrosigmoid elliptical craniectomy is exposed. Under microscopic view, the dura is opened, and the CSF drainage allows for a natural surgical corridor to the cerebellopontine angle.

**2:03 Trigeminal Combing.** Microsurgical exploration of the entire length of the trigeminal nerve rules out significant vascular compression. The root entry zone appears pale and malacic. The trigeminal nerve is longitudinally divided with a microknife along its fascicula in the root entry zone area, allowing for an accurate interfascicular cleavage plane. Interfascicular neurolysis is performed with a blunt round dissector. The trigeminal fascicula are now exposed and separated. Capillary bleeding is tolerated, and spontaneous hemostasis is obtained by simple irrigation.

**3:01 Postoperative Course.** The postoperative course was uneventful. A trigeminal sensory deficit was evident in maxillary and mandibular branches, without masticatory disturbances. Pain immediately disappeared. The sensory deficit spontaneously recovered in the following months. The patient is pain free without medications at a 1-year follow-up.

**3:26 Trigeminal Combing: Indications.** Trigeminal combing is indicated for drug-resistant trigeminal neuralgia in multiple sclerosis patients when there is no intraoperative evidence of vascular cross-compression.^2,4,5^ In addition, it is applicable to any trigeminal neuralgia patient without intraoperative evidence of neurovascular compression.^5,6^

## Author Contributions

Primary surgeon: Ferroli. Assistant surgeon: Vetrano. Editing and drafting the video and abstract: Vetrano, Ferroli, Acerbi, Raccuia, Schiariti, Chiapparini, Broggi. Critically revising the work: Vetrano, Ferroli, Acerbi, Raccuia, Schiariti, Chiapparini, Broggi. Reviewed submitted version of the work: Vetrano, Ferroli, Acerbi, Raccuia, Confalonieri, Chiapparini, Broggi. Approved the final version of the work on behalf of all authors: Vetrano. Supervision: Ferroli, Acerbi.
